# Older patients are at increased risk for renal posttransplantation diabetes mellitus

**DOI:** 10.1186/1758-5996-7-S1-A99

**Published:** 2015-11-11

**Authors:** Ana Laura Pimentel, Priscila Aparecida Correa Freitas, Joíza Lins Camargo

**Affiliations:** 1 IPA, Porto Alegre, Brazil

## Background

Posttransplantation diabetes mellitus (PTDM) is a metabolic complication related to the use of immunosuppressive medication after renal transplant. Identifying patients at highest risk of PTDM would help clinicians in their management, once PTDM is associated with higher number of graft rejection and death in the long-term.

## Objectives

The aim of our study was to determine the risk factors associated with PTDM development at the fourth month after renal transplantation.

## Materials and methods

All patients without diabetes who underwent renal transplant at a University Hospital between July 2012 and June 2015 were included. PTDM was diagnosed according to current ADA criteria at four months after transplantation. Poisson regression with robust standard errors was performed with PTDM as dependent variable and the possible risk factors under study (age, sex, type of donor, immunosuppressive type, family history of DM, pre-transplant BMI and fasting plasma glucose) as independent variables. P-value <0.05 was considered as statistically significant.

## Results

One hundred fifty-eight patients were included in the study and 24.1% had PTDM diagnosed at four months after transplantation (50.6% men, mean age 46.1±13.1 yrs.). The only factor associated with PTDM in our cohort was age (p<0.001; relative risk 1.064 [1.033-1.095]). Each one year increase in age was associated with 6.4% higher risk for PTDM.

## Conclusions

Our cohort showed high incidence of PTDM at four months after renal transplantation. Older patients should be counseled of their incremental risk for PTDM, since the proper management can reduce the risk of graft rejection and death in the long-term.

